# Inner and Outer Contextual Factors Impacting Mental Health and Criminal Legal Cross-Systems Collaborations

**DOI:** 10.1007/s10488-025-01474-7

**Published:** 2025-09-18

**Authors:** Stacey L. Barrenger, Leslie L. Wood, Natalie Bonfine

**Affiliations:** 1 Department of Psychiatry, Northeast Ohio Medical University, 4209 St. Rt. 44, PO Box 95, Rootstown, OH 44272 USA; 2 Department of Sociology and Criminology, Kent State University, Kent, OH USA

**Keywords:** Cross-systems collaborations, Criminal legal system, Mental health system, Serious mental illnesses, EPIS framework, Collaboration strategies

## Abstract

People with serious mental illnesses continue to be overrepresented within the criminal legal system despite multiple diversion and reentry intervention efforts. Engaging in a coordinated systems-level approach to this problem has increased, as mental health criminal legal cross-systems collaborations, like Stepping Up and Sequential Intercept Mapping, proliferate across the United States. Despite their proliferation, little is known about how these cross-systems collaborations operate, including what factors are present and how these factors help or hinder group effectiveness. Stakeholders engaged in mental health criminal legal cross systems collaboration participated in focus groups and in-depth interviews. Using the Exploration, Preparation, Implementation, and Sustainment framework to guide the analysis, findings showed that inner, outer, and bridging factors feature predominately in cross-systems collaborations. Inner and outer contextual factors like leadership, values, funding, and data accessibility are important to their operations. Additionally, bridging factors of purveyors (engaging in technical assistance) and systems-level collaboration strategies (cross-training, sequential intercept mapping, and data sharing) were important to supporting sustainability. Future research should investigate which systems-level collaboration factors are tied to the implementation of new practices, programs, and policies which in turn may improve the behavioral healthcare system and health outcomes for people with serious mental illnesses.

Despite significant efforts to reduce their presence in the criminal legal system, people with mental illnesses continue to be overrepresented within jails, prisons, and probation/parole (Bronson & Berzofsky, 2017; Van Deinse et al., 2019a, 2019b; Wolff et al., 2014). Once in contact with the criminal legal system, they spend more time incarcerated and are more likely to return to jail or prison (Comartin et al., 2020; Eno Louden & Skeem, 2011). Individuals with serious mental illnesses (schizophrenia, bipolar disorder, and major depression) and criminal legal system involvement are also more likely to have a substance use disorder and experience homelessness and trauma, leading to a complex array of needs requiring multiple services and supports. The challenge of meeting these needs poses a systems-level problem as it is costly and burdensome for the criminal legal and mental health systems. Cross-systems collaborations (CSCs) between the mental health and substance use services (i.e., mental health system) and the criminal legal system aim to improve outcomes for people with mental illnesses involved in the criminal legal system through coordination of services across multiple systems of care. Collaboration across systems can result in the establishment of new, or a revision of existing, practices, programs, and policies, exemplified by cooperation and coordination from agencies across multiple systems (Mackey et al., 2024), with the goal of reducing the involvement of people with mental illnesses in the criminal legal system.

## Mental Health and Criminal Legal Cross-Systems Collaborations

Mental health and criminal legal CSCs are a type of cross-sector collaborations aimed at improving community health (Mattessich & Rausch, 2014), these collaborations can range from informal practices such as local police departments notifying behavioral health agencies when they have contact with a person experiencing a mental health crisis to more formalized efforts such as the establishment of a specialized treatment court. Likewise, the level of stakeholder involvement, amount of coordination of services, and cooperation between and across different entities can vary. Historically, the creation of mental health and criminal legal CSCs existed on an ad hoc basis to establish a specific practice, but more recently, CSCs have been tasked with the broad goal of reducing contact with the criminal legal system for people with mental illnesses, which can take the form of a variety of practices, programs, or policy changes (Mackey et al., 2024). One effort is based on the Sequential Intercept Model (Munetz & Griffin, 2006) which highlights the several points at which individuals can be diverted from criminal legal contact and into community-based mental health services; another effort is the National Stepping Up Initiative focused on reducing the population of individuals with serious mental illnesses in jails.

The Sequential Intercept Model is used to highlight the points at which individuals with serious mental illnesses have contact with the criminal legal system (Munetz & Griffin, 2006). These intercepts become potential targets of policy or practice like pre- or post-booking jail diversion programs (Case et al., 2009) at Intercepts 2 & 3. The *Sequential Intercept Mapping and Taking Action for Change* workshop (referred to here as Sequential Intercept Mapping), developed by Policy Research Associates, is a cross-systems approach to strengthening local strategies to implement core services addressing behavioral health, criminal risk, and social factors for individuals with mental illnesses and criminal legal involvement (Griffin et al., 2015a, 2015b). In Ohio, Sequential Intercept Mapping is a facilitated, action-planning workshop where community members work together to evaluate gaps in the system across intercept points and identify priority areas for intervention (Ohio Criminal Justice Coordinating Center of Excellence, 2025). During the Sequential Intercept Mapping exercise, trained facilitators guide community members through a discussion about opportunities and resources to divert individuals away from the criminal legal system and to link individuals to treatment and services, resulting in an Action Plan for Change. Community members then form smaller working groups to carry out the work identified during Sequential Intercept Mapping workshop. In 2016, the United States Congress recommended the Sequential Intercept Model and Sequential Intercept Mapping to increase public safety by facilitating collaborations among behavioral health and criminal legal systems partners (Twenty-First Century Cures Act of 2016, Public Law 114–255, Title XIV, Subtitle B, § 14,021).

The Stepping Up Initiative is a related community-based initiative that encourages mental health and criminal legal system collaboration to reduce the number of people with serious mental illness in local jails (The Stepping Up Initiative, 2020). Strategies for reduction include better identification of people with mental health and co-occurring substance use disorders who are incarcerated and then implementing programs, policies, or practices to reduce their presence in the jail. Achieving Stepping Up goals requires compiling and monitoring data, which necessitates agencies sharing information and tracking metrics across the criminal legal and mental health systems. Nationally, approximately 580 counties have passed resolutions to engage in The Stepping Up Initiative and in Ohio, 62 of 88 counties are participating (The Stepping Up Initiative, 2025).

With the widespread adoption of Stepping Up and Sequential Intercept Mapping, communities are moving beyond efforts to implement individual programs specific to a single agency or organization, like crisis intervention team training or specialized dockets, to identifying programs and practices across multiple intercepts of the criminal legal system and within an expanded behavioral health system (Aarons et al., 2014b; Bonfine & Nadler, 2019; Mackey et al., 2024). As such, the work of cross-systems collaborations has become broader and more complex, requiring collaboration among more systems players. Several programs may be at different stages of development across Intercepts simultaneously, and the CSCs are often tasked with implementing and coordinating these multiple initiatives. In addition to bringing together key personnel or agency representatives, CSCs are also charged with building ties to other community groups, task forces or collaborations (e.g., suicide prevention task force, homelessness coalitions). While there remains a need for research evidence to support implementation of single programs at the interface of the behavioral health and criminal legal system, less is known about the synergistic effects of multiple programs at system- or individual-levels (Bonfine et al., 2023; Swanson et al., 2023) or what it takes for CSCs to successfully develop, implement, and sustain multiple programs.

Both Sequential Intercept Mapping and Stepping Up bring stakeholders from multiple systems together and these partnerships rely on local leadership, resources, and capacity to negotiate varied interests and motivations. Research and evaluation on Sequential Intercept Mapping found improvements in collaboration, cross-systems education, and improving programming and services (Bonfine & Nadler, 2019; Griffin et al., 2015a, 2015b). A national study of Stepping Up found that a well-developed health care infrastructure was a better indicator of Stepping Up participation than crime or socioeconomic factors (Ramezani et al., 2023), suggesting that counties with more resources are more likely to engage in these initiatives. While this research shows mental health-criminal legal CSCs may be beneficial to improving coordination of services, little is known about how these collaborative groups operate or the contextual factors that support or impede their development and sustainment. Stepping Up and Sequential Intercept Mapping collaborative groups have become increasingly responsible for planning and implementing multiple programs and approaches simultaneously with little guidance on how to best coordinate or sustain collaborative efforts (Mackey et al., 2024). Without such guidance, communities may be wasting limited personnel and financial resources, and their efforts may not be specific or targeted enough to address the needs of their community.

## Cross-Systems Collaborations

Mental health and criminal legal CSCs are subject to not only group and organizational dynamics but also are tasked with aligning interests and actions across a diverse group of members with differing priorities, which can be challenging (Bonfine & Nadler, 2019). For example, the criminal legal system is primarily focused on public safety, whereas the mental health system prioritizes well-being. While these are complimentary services within a community, their different primary goals can be at odds when developing cross-system initiatives. For example, as law enforcement may be more focused on a goal of public safety and may not be trained to recognize public health and wellbeing as an aspect of their roles (Van Dijk et al., 2019). In reviewing research on cross-sector collaborations, including their own work, Bryson and colleagues (2015) developed a consolidated framework identifying major concepts associated with collaborative groups, which included the contextual factors, process components, and structural factors endemic to cross-sector collaborations. General antecedent conditions such as the resources available and the environment, along with initial conditions, drivers, and linking mechanisms lay the foundation for these types of collaborations. For example, external factors such as a supportive environment, including political and socioeconomic factors (Nagorcka-Smith et al., 2022) can impact the work of collaborative groups. Within these groups, collaborative processes and structures like trust and commitment, a shared understanding of the problem, communication, leadership, and the development of norms and practices of engagement are important factors (Bryson et al., 2015; McGuire & Silvia, 2009). Yet, endemic tensions such as flexibility vs. stability and self-interest vs. collective interest are common and an inability to balance interests between members can stall the efforts of these groups (Bryson, et al., 2015; Mosely, 2021; Thomson & Perry, 2006). As mental health-criminal legal CSCs proliferate across the country, it is imperative to better understand both the structure and function of these multi-level organizational entities.

## EPIS Framework

Drawing from organizational literature and originating from implementation science, the Exploration, Preparation, Implementation and Sustainment (EPIS) framework (Aarons et al., 2011; Moullin et al., 2019) can be a useful guide to understanding both the structure and the function (i.e., organizational dynamics) within CSCs. As mental health-criminal legal CSCs attempt to implement multiple programs, policies, and practices simultaneously, the EPIS framework can enlighten both the complex processes involved and how they impact innovative implementation. The EPIS framework describes four phases of the implementation process for evidence-based practices (EBPs). The Exploration phase encompasses the identification of a clinical problem and begins identifying appropriate EBPs, taking into consideration the need for adaptation to the local environment. In the Preparation phase, adopters identify potential barriers and facilitators of implementation, along with developing a plan to guide the Implementation phase where the EBP is initiated and monitored. The Sustainment phase ensures the necessary supports are in place, so the EBP remains an effective intervention.

The framework also includes contextual factors that support or hinder implementation of EBPs. The outer context, comprising the external environment, features factors such as funding/contracting, workforce, state-level policies, inter-organizational relationships, and community characteristics. Whereas the inner context comprises the organizational environment supporting the innovation or EBP being implemented, and the factors that contribute to the direct operations of the EBP. Such operations include leadership, support structures, group values and goals, communication and information sharing, problem solving strategies, relationships, and teamwork (Moullin et al., 2019). The inner and outer contexts of this framework are connected by bridging factors, like community and academic partnerships, and inter-organizational practices, that facilitate the implementation of the EBP. The innovative factors in the EPIS framework are the characteristics of a specific EBP and how it fits with the organization or providers.

The EPIS framework is comprehensive in identifying conditions that affect implementation across multiple levels and contexts, while also describing the process or stages of implementing innovative practices. The EPIS framework has been used to study implementation of EBPs in a variety of health and social service-related fields, including complex strategies that support multiple evidence-based process improvement efforts (Moullin et al., 2019) and CSCs between child welfare and behavioral health systems (Bunger et al., 2024). As a process and determinant framework, EPIS has been flexibly applied to teams within implementation settings (McGuier et al., 2023) and in health policy research (Crable et al., 2022).

Similarly, EPIS has the potential to guide implementation of mental health-criminal legal system CSCs because it encompasses the multi-level contextual factors inherent when working with stakeholders across multiple systems. Mental health criminal justice CSCs, like Stepping Up and Sequential Intercept Mapping groups, often engage in systems alignment work to address multiple priorities, values, or operations among systems players (Bonfine & Nadler, 2019), especially during the exploration and preparation phases prior to implementation of any policy or practice. Because of this critical planning role, the inner context of the EPIS framework applies to the CSC itself, rather than any single implementing agency, as the organizational body that is tasked with implementing one or more innovations (or systems-level policies, practices or programs). While the function of mental health criminal legal CSCs can be explicated by the inner context, which has the potential to highlight the factors important to forming and sustaining a collaborative group, understanding the outer context may uncover local, state, and federal policies that potentially impact the function of the CSC or its ability to make progress and achieve goals. Furthermore, identifying bridging factors may point to collaboration strategies that aid in systems alignment needed for cross-systems implementation (Chaung et al., 2024).

This study applies the EPIS framework to an analysis of CSCs to address the following questions: 1) What are the outer context, inner context, and bridging factors of cross-systems collaborations? and 2) How are these factors perceived to facilitate or impede cross-systems collaborations?

## Methods

### Study Setting and Framework

The current study applied the Exploratory, Preparation, Implementation, and Sustainment (EPIS) framework to focus groups and individual interviews from a pilot study of mental health-criminal legal CSCs in Ohio. The focus groups and interviews were held as part of a larger study to co-develop and pilot a self-evaluation toolkit intended to provide technical assistance and guidance to newly formed cross-systems collaborations or CSCs. In the parent study, focus groups and in-depth interviews were conducted to elicit best practices and challenges associated with mental health criminal justice cross systems collaborations. Information from the interviews and focus groups was used to develop systems collaboration and data inventory toolkits. Participants were invited to co-design session to review the toolkits and provide feedback and design suggestions. Since the interviews and focus groups also contained a wealth of information on the structure and function of mental health and criminal legal CSCs, we engaged in this additional analysis to better understand this rarely examined collaborative group.

### Recruitment and Sampling

We recruited systems collaboration leaders and experts by experience across Ohio to participate in focus groups to understand their attitudes, knowledge, and beliefs about cross-systems collaboration and to identify common and consequential barriers to measuring processes and outcomes related to their collaborative efforts. Notices about the study were included in the state’s Stepping Up newsletter and emails were sent to individuals who had ever participated in Sequential Intercept Mapping workshops since 2013. These outlets have broad circulation in the state, as over two thirds of the counties in Ohio have passed a Stepping Up resolution, and over 1,000 people have participated in over 40 Sequential Intercept Mapping workshops since 2013. Both emails and the newsletter announcement included a link to a Qualtrics survey that screened individuals for eligibility, identified their role within the CSCs and collected basic professional data. If participants met eligibility criteria (over 18 and currently involved in a CSC), they were linked to a page to sign up for a focus group. Participants were directed to a focus group aligned with their role in the group. The collaboration leader group included eight individuals, three reported working in behavioral health agencies and five worked in the criminal legal system; the expert by experience group included three individuals who reported being advocates, family members, or a person with lived experience. Participants were grouped by their roles as we thought their perspectives on and experiences with CSCs may differ substantially and we did not want potential differences to dominate a focus group discussion.

### Data Collection and Analysis

Our sample size for this analysis is 11 participants, each representing 11 different CSCs from across the state. We intended to complete all data collection via focus groups because we thought the interactive nature of a focus group would produce richer data. However, coordinating times when interested participants could be available proved difficult. Therefore, we also conducted individual interviews with interested participants who could not attend a scheduled focus group. We conducted two focus groups (6 participants) and completed five individual interviews between May and August 2022. Each participant attended either one focus group session or participated in an interview if they were not available to attend a focus group. The first and third authors participated in all the focus groups and interviews, with one person taking the lead in asking questions from an interview guide while the other person took notes and asked clarification questions, if necessary. The same interview guide was used for both focus groups and interviews and for both collaboration leaders and experts by experience. Topic areas included general attitudes about mental health criminal legal collaborations, the collaborative process, barriers to progress, and data and self-evaluation practices. Study procedures were approved by our IRB. Both the first and third authors have many years of experience conducting research at the intersection of the mental health and criminal legal systems. The third author has also previously engaged in research and concucted evaluations focused on cross-systems collaborations, specifically Sequential Intercept Mapping.

Transcripts from the focus groups and interviews were analyzed using the Sort & Shift, Think & Shift methods developed by Maietta and colleagues (2021). This method includes several iterative processes of “diving in” and then “stepping back” from the data, which allows both a close examination of the data and the ability to form broader connections across the data. To dive in, the first author created an episode profile for each focus group or interview, which included a quotation inventory, a cluster diagram of the most relevant quotations, and a memo outlining why each quotation was captured and its relevance to the research questions. These episode profiles created a vertical (or in-depth) understanding of each focus group or interview. We then stepped back by building a visual inventory of the quotations across all episodes by assigning quotations to topics, mining data from the episode profiles to identify core themes by seeing how ideas work together within and across episode profiles and topics, thereby creating a horizontal understanding of the data. We employed analytical triangulation, peer debriefing, and created an audit trail to strengthen the credibility and dependability of our findings, ensuring that core themes are represented both within and across the data set (Padgett, 2008). After identifying core themes, the first and last author met several times to review core themes and discuss their relevance to the EPIS framework. These discussions led to an understanding of how EPIS concepts operate within mental health and criminal legal CSCs.

## Results

### Cross-Systems Collaborations and the EPIS Framework

The EPIS framework guides the organization and the presentation of our results, especially in understanding the outer context, inner context, and bridging factors of CSCs. In line with the first research question, many aspects of the EPIS framework (see Table 1) are present in mental health and criminal legal CSCs. Multiple concepts related to the outer context, inner context, and bridging factors were reflected in participants’ responses. Within EPIS the inner context often refers to the organizational characteristics of the entity implementing the EBP; however, within our conceptualization, the inner context refers to the characteristics of the CSC—the entity engaged in systems alignment to develop and implement systems-level practices, programs and/or policies. Therefore, the outer context refers to the external factors that influence the CSC’s ability to carry out its mission. Bridging factors include both the purveyors and intermediaries along with the collaboration strategies that CSCs employ at the systems level to facilitate systems alignment. Within the outer context, participants described state leadership, the National Stepping Up initiative, funding, and emerging best practices, but there was no mention of state policies, clients/services, or advocacy as related to participants’ CSC efforts. Inner context factors present in our data included leadership, vision & values, stakeholders, intraorganizational cooperation, data capacity and resources, and funding. For the bridging factors, collaboration strategies such as sequential intercept mapping, cross-systems training, and data sharing were identified by participants, along with the role of purveyors providing technical assistance. Many of the outer, inner and bridging factors identified in the EPIS framework are closely aligned with the concepts highlighted by our participants.

**Table 1:** Identified Outer, Inner & Bridging Factors of Cross-Systems Collaborations

*Outer cvontext*	State leadership (behavioral health services)
	National Stepping Up
	Funding/grants
	Emerging best practices
*Bridging factors*	Purveyors/ intermediaries (e.g., TA Centers)
	Collaboration Strategies
	SIM
	Cross-systems training and education
	Data sharing
*Inner context of CSC*	Leadership
	County Level
	Collaboration Level
	Collaboration Characteristics
	Shared Vision & Values
	Intra-organizational cooperation
	Stakeholder Characteristics (voluntariness)
	Data capacity & resources
	Funding

### Facilitators and Barriers to Mental Health Criminal Legal Cross-systems Collaborations

The second research question explored the ways in which the outer context, inner context, and bridging factors were perceived to facilitate or impede cross-systems collaborations. These findings show the factors that are critical to CSCs’ success or stagnation.

#### Outer Context

Outer context factors that supported or hindered mental health-criminal legal CSCs include leadership at the state level, national initiatives, funding, and emerging best practices in the field to address criminal legal system involvement of people with mental illness. Participants did not specifically mention state policies, clients/services, and advocacy in the focus groups or interviews.

**State Leadership & National Stepping Up.** State leadership is critical to raising awareness around CSCs in Ohio such as Stepping Up and Sequential Intercept Mapping. Stepping Up is a national effort and is administered through the state agency for mental health and addiction services in Ohio, which maintains a website with information, resources and links to the national Stepping Up website (The Stepping Up Initiative, 2025). There is a statewide Stepping Up steering committee headed by a retired Ohio Supreme Court justice. Additionally, a statewide Center of Excellence partially supported by state monies, provides Sequential Intercept Mapping to Ohio counties (Ohio Criminal Justice Coordinating Center of Excellence, 2025).

Participants agreed that state leadership in supporting initiatives like Sequential Intercept Mapping and Stepping Up brought people together, refocused previous collaborative efforts, and helped communities set goals. As one community leader related, “I'm not sure if it [multiple initiatives within the county in recent years] would have come together as fluidly as it did without people learning from the Stepping Up experts across the state” (Collaboration Leader 1, lines 126–127). State leadership through endorsement of these activities prompted communities to form mental health and criminal legal CSCs, as this community leader stated, “And I just think Stepping Up was really helpful to, like I said, re-energize and bring the leadership and the champions back together in one room and refocus. And I think that was really instrumental for us” (Collaboration Leader 1, lines 75–76). Even in communities where agencies commonly work together, participating in Sequential Intercept Mapping provided a focus for the group leading to tangible change. As this community leader shared,

You know, everybody kind of knew everybody but we didn't necessarily always sit in the same room and talk about the topics collaboratively. Um, I think we have a pretty collaborative community but, but this really helped us, you know, map out the steps, which is what it's supposed to do so. There were a number of things that…that changed. (Collaboration Leader 1, lines 88-91)

**Funding.** Despite strong state and national leadership that facilitated the initiation of mental health-criminal legal CSCs in Ohio, to our knowledge there is no state funding dedicated to supporting the collaborations. The state mental health agency has provided funding for specific mental health/criminal legal system initiatives and communities have utilized these funds to implement a variety of programs targeting one or more intercepts, or specific populations. For example, small “mini-grants” are made available to support costs associated with Crisis Intervention Team training (National Alliance on Mental Illness Ohio, 2025). Yet there is no state-level funding to support the overarching work of the CSCs directly. Thus, the complex task of populating, convening, training, and sustaining a diverse and representative group of stakeholders to develop overarching, cross-systems goals does not have dedicated funding. Often groups such as task forces, advisory boards or planning committees form around a specific project or an issue adjacent to mental health-criminal legal CSCs that has received state funding. Such projects include improving crisis response services, suicide prevention, or assisting populations at risk of overdose or death.

For example, Ohio is an opioid abatement state and has money dedicated to addressing opioid addiction, yet this is not the target population of mental health-criminal legal CSCs. Recognizing this disparity caused frustration for one community leader, “There's dollars that are available to help this population [opioid use disorder] and yeah, we don't want to discount that. But there's also a much broader population over here, you know?” (Collaboration Leader 4, lines 189–191). The lack of dedicated funding and continued engagement with policymakers posed challenges for community leaders, as discussed by one participant, “Having access to policy, legislators, and money is also really important. You can’t get some of this work done without [those things]” (Collaboration Leader 1, lines 667–671). Without dedicated funding for personnel or seed money for projects, many CSCs struggle to progress beyond an initial start-up phase or expand their focus beyond the initial issue.

**Emerging Best Practices.** Due to the lack of robust evidence on practices that keep people with mental illnesses out of jails and prisons, both Stepping Up and Sequential Intercept Mapping operate on encouraging counties to adopt “emerging best practices” and policies. The dearth of reliable, evidence-based interventions in this area of practice results in a disparate array of promising practices that mental health-criminal legal CSCs may implement. Within this context, participants mentioned a variety of practices that their CSCs had implemented (e.g. monitoring jail or hospital data, implementing jail in-reach services, or notifying mental health agencies about law enforcement encounters) that do not necessarily rise to the level of evidence-based. However, these practices encourage systems alignment by providing information across systems or engaging in systems-level coordination. Additionally, there are a wide array of practices and approaches across the sequential intercept model that are being planned and/or implemented simultaneously, making it difficult for mental health-criminal legal CSCs to evaluate which practices, programs, or policies might be the most promising for their local context. Stakeholders are aware of the importance of collecting and analyzing data to demonstrate program effectiveness and to guide planning for implementing programs, yet purposeful data collection and analysis remains a challenge. One community leader described how in their community they did not have a comprehensive command of their data but recognized the cultural shift towards expectations of data to support new ideas or initiatives (Collaboration Leader 1, lines 369–372).

#### Inner Context

Inner context factors were also perceived as facilitating or impeding CSCs (research question 2). These factors were systems collaboration leadership, collaboration characteristics, stakeholder characteristics, cooperation, data capacity and resources, and local funding.

**Systems Collaboration Leadership.** Leadership, at two levels, is very key to mental health-criminal legal CSCs. County-level leadership, such as county commissioners, elected officials (e.g., Sheriff, judges, mayors), or senior administrator for behavioral health services (e.g., mental health board directors), is important in bringing together representatives across the many systems and organizations. Leadership presence plays a key role in generating buy-in to the process, embracing the change initiative component of the collaboration, and advocating for policies and funding. Leadership at the collaboration level is important to coordinate the activities of the collaborative group, communicate across the collaborative group and up to county leadership, and provide direction and resources for stakeholders.

***County Leadership****.* County-level leadership is important in getting mental health – criminal legal CSCs started as they have the leverage to bring multiple systems players together, as people show up when a judge or county administrator calls for a new initiative. An expert by experience group member explained this as, “So, if you have somebody like that, again, that influencer. That person who can make them pull the levers and people have to follow” (Expert by Experience 1, lines 486–488). The presence of active county-level leadership also facilitates engagement with the process. They often have the power to jump-start collaborations or sustain them by providing funding for the collaboration or advocating for policy changes to support the collaborative mission. Communities without this type of engaged leadership faltered, as one collaboration leader stated,

I mean there really does need to be a driver, and you know somebody with the capacity and interest in in this work in order to make things happen, because if you, if you don't make any noise about it, it just completely stalls in the water. (Collaboration Leader 3, lines 67-69)

***Collaboration Leadership***. Having a coordinator position for mental health-criminal legal CSCs is critically important to sustain the momentum garnered from county leadership buy-in. Many participants observed from their own experiences that without someone setting meetings and agendas, facilitating communication, and providing some accountability to members and workgroups, these types of groups falter after the initial start-up, as pointed out by this community leader, “Since we have been doing this work, we identified that it really does need a designated person behind the scenes to ensure that everyone is meeting, that the work is getting done” (Collaboration Leader 2, lines 127–129).

It is also important that this coordinator have sufficient time, resources, and support to carry out this work. Many coordinators occupied multiple roles and did not have sufficient time to properly manage the collaboration. While in these instances collaborative efforts did not stop altogether, they were hampered by the lack of attention and time devoted to it, as this participant reiterated, “So, you know, again it's, it comes down to workforce and capacity. I sure wish that we could bolster these initiatives with a paid position…It's worth its weight in gold, but we just don't have that, it's unfunded” (Collaboration Leader 3, lines 94–96).

The coordinators of mental health-criminal legal CSCs are both the heart and brains of these projects as they are committed to the collaborative process, attempt to understand the many different roles played by systems actors, intuitively understand the needs of the community, keep people engaged and focused on the task, and have knowledge about how to begin addressing data needs, while also promoting programmatic changes that can benefit their communities. While they have limited power, per se, they are powerful players in these CSCs. They are the ones who keep things going and have both a bird’s eye view and frontline knowledge of what needs to be done. However, this leadership role can also be taxing. As this collaboration leader shared, “To be on the forefront constantly is challenging; I think it can be exhausting for the coordinator, who is doing a lot of work behind the scenes” (Collaboration Leader 1, lines 518–521). For CSCs without a designated coordinator, this behind-the-scenes work is rarely executed.

**Vision & Values**. Many participants identified a purpose for their CSCs and felt that group members were working towards that same goal. Furthermore, some participants indicated an awareness that their identified goal was a shared problem that could not be solved by any one system and that a collective effort, across multiple systems, was the only way to effect change. A shared purpose and perspective helped stakeholders to shift from working independently to focusing on collaborative solutions, as this expert by experience shared,

But I, like, think the very fact that we're all getting together and talking about things…if nothing else, there's been a slight cultural shift in terms of, we're not all working in our own silos anymore. We really do have to start coming together and seeing this as a holistic problem. (Expert by Experience 2, lines 143-145)

An expert by experience participant observed that identification of a shared goal allowed the group to focus on solutions, “there's kind of an acknowledgement what we've been doing hasn't worked and so now I think there's more discussions around solutions to the problem. So we're not trying to identify the problem; we kind of know what problems are” (Expert by Experience 1, lines 324–326).

CSCs without an identified shared problem or population to address struggled to sustain their efforts or produce any meaningful outcomes as recounted by this expert by experience, “for the first four or five years that our Stepping Up committee was meeting, it was a huge waste of time. Until the point where we have most– many of our members had dropped out because we weren't making any progress” (Expert by Experience 2, lines 106–109). However, this CSC changed its course by engaging in sequential intercept mapping, which allowed the group to “…have someone who is in charge of a priority. It has kept the momentum going, and it’s given our Stepping Up committee something to focus on too” (Expert by Experience 2, lines 126–128). If stakeholders do not see the work of the group having a potential impact on their population, they may stop showing up and being part of the process.

**Stakeholders.** Stakeholders are very important in this process as they fostered cooperation among system players, showed commitment to solving seemingly intractable problems, and developed professional relationships that extended beyond the collaboration group. While many stakeholders spoke of the voluntary nature of serving on CSCs with limited time and capacity to attend to the work, they almost exclusively spoke of their fellow stakeholders in positive terms, often citing them as the strongest component of their current collaborative work. One collaboration leader shared that she perceived collaboration participants as interested, showing up, and “bringing something to the table” (Collaboration Leader 4, line 279). Furthermore, some participants thought that competition between providers evaporated once stakeholders understood the scope of the problem along with understanding that they shared clients across multiple systems.

Additionally, stakeholders serving on cross-systems collaborations do so in addition to their ongoing jobs and responsibilities, causing many participants to identify themselves as “volunteers” or “volunteering my time” to the CSCs. A perspective of voluntariness was stated by both collaboration leaders and experts by experience, “But it's a whole other thing to do anything within [the collaborative group], because no one's getting paid to do anything so it's all volunteer, and it can be a lot of work” (Expert by Experience 2, lines 132–134). Seeing their participation as voluntary made it easier for participants to have less commitment to the collaborative process and outcome, as the work of Stepping Up or Sequential Intercept Mapping is a lower priority on their very full calendars.

**Inter-organizational Cooperation.** Participants described fellow stakeholders as potential barriers to the work, especially if they do not fully understand the need for the CSC. Some mentioned stakeholders feeling threatened by change, or concerned about competition from fellow providers, or not understanding legalities around HIPAA consultations. Turnover among stakeholders was also identified as a problem, in that either people would stop showing up and did not send a replacement for them or the replacement may show up but was not briefed on the purpose of the group. In one instance, an expert by experience stakeholder mentioned that she refrained from participating fully in her group because she did not want to go against a funder of her agency, “So, [I had] a funder…in my group, I was trying to be careful not to overstep or correct that person, so I just kind of kept some comments to myself” (Expert by Experience 3, lines 130–132).

Lastly, some participants revealed that some stakeholders may be resistant to moving from working in silos to collaboration as this requires change. As observed by this expert by experience, “I mean, you know, a lot of times we're proposing change. Change to the system, change to anything, and the obstacle would be, as some people immediately…rise up in opposition to change” (Expert by Experience 1, lines 281–284) When this stakeholder who is resistant to change is a critical part of the CSC, like a county jail or behavioral health provider, it becomes difficult for the group to progress.

**Data Capacity & Resources**. Data was a common concern among all participants, specifically challenges with data management, data storage, data access, and data analysis. Working with data is a stumbling block due to the lack of capacity within communities to address data needs for CSCs. Participants identified a need to hire data managers but noted lack of resources to or an inability to devote staff to data management. An expert by experience acknowledged,

[There is] a need to have someone who understands data collection, who could help us. You know, there's data out there…It's just that we don't know how to access it or how to, you know, make apples and oranges come together, to look like something…and none of us have that skill set. (Expert by Experience 2, lines 327-331)

One community leader reported that data barriers in his community included both a lack of understanding of the importance of data monitoring and a stakeholder who did not collect or track any data,

You know [they are] kind of scoffing at the idea of collecting any kind of data…I mean historically, [they] just don't do that. And so…getting everybody on the same page about the importance of establishing a baseline and going from there…just collecting data [is difficult]. (Collaboration Leader 3, lines 356-359)

Some communities focused narrowly on a specific data task, like data sharing across multiple systems, which proved difficult to accomplish. When they could not make this happen, they gave up trying to share or manage any data collaboratively. Conversely, some participants reported that their communities responded flexibly and logically to data challenges by sharing what data they had, connecting new goals and objectives to data outcomes, or merely identifying which agencies have what type of data at their disposal. For example, one collaboration leader described how their data subcommittee did a mini mapping of the data in hopes of better communication and analysis, but noted how it is overwhelming to figure out (Collaboration Leader 1, lines 271–279). Whereas another CSC used anecdotal information to show progress despite the lack of metrics, “when we can’t collect quantitative data; we can still put some good stories out there…sharing success stories makes Stepping Up worth it and people think they should continue participating” (Collaboration Leader 1, lines 268–274).

**Funding.** As discussed, both Stepping Up and Sequential Intercept Mapping are initiated in communities through local leadership and no funding stream exists to support these efforts. Therefore, many communities attempt to engage in CSCs without funds to support the work. A participant shared that this lack of funding is a threat to progress, “There's no money behind it, which is a huge problem. So, a lot of the things that we wanted to do, like starting to collect data, we just didn't have the finances to do it, we couldn't” (Expert by Experience 2, lines 115–118). Higher-resourced communities who see the value of developing cross-systems strategies divert money from other projects to fund staff to coordinate and lead group activities. Communities without dedicated and paid leadership struggle to establish vision and goals, meet regularly, and develop new initiatives.

#### Bridging Factors

There were multiple bridging factors identified in the data and these linkages between the inner and outer contexts mostly helped to facilitate the work of CSCs.

**Purveyors and Intermediaries: Technical Assistance.** To become a Stepping Up county in Ohio, local leaders must pass a resolution declaring the county a Stepping Up county and identify group members (The Stepping Up Initiative, 2025). Purveyors, providing technical assistance, were especially helpful in spearheading sequential intercept mapping, which promotes representatives from all sectors who, in a facilitated discussion, map community services across the sequential intercept model. The Sequential Intercept Mapping exercise highlights gaps in the system, as participants jointly decide on which gaps to prioritize by identifying goals and forming work groups. Sequential Intercept Mapping and Stepping Up efforts were synergistic within these communities, and participants viewed both initiatives as critical to getting multi-sector partners with different perspectives working towards shared goals. Community Leaders also shared that Sequential Intercept Mapping brought people together in a process that seemed more solution-focused rather than assigning blame to agencies for gaps in the system. The Sequential Intercept Mapping approach also allowed individuals an opportunity to begin to understand the roles of other system actors and how they could possibly work together across silos. One community leader expressed these phenomena,

I think, for us, the sequential intercept mapping…really helped. [They] Kind of walk us through [the process and] you're not pointing fingers, [instead] you're looking at solution-oriented approaches. And, then the process itself helps everybody along that continuum understand what roles other people have to play. So, I think that, you know, that the process itself helps with that…And then you, you know, you put people in the same room and, you know, eventually, they start talking to one another and recognize each other's role and strength. (Collaboration Leader 1, lines 500-506)

For an expert by experience, participating in Sequential Intercept Mapping helped their CSC become unstuck by providing specific goals,

For the first five years we didn't have any goals. It's like we'd come to these meetings, and we would just spin our wheels, and nothing was moving. Nothing was happening. It’s like, why are we doing this? But once we had the Sequential Intercept Mapping and were able to identify the areas that we wanted to look at, and we at least now have some identified priorities, some areas to put our efforts and our energies-- so that's been huge. (Expert by Experience 2, lines 312-316)

Targeted technical assistance provided a catalyst for communities to begin or renew their cross-systems efforts. However, technical assistance beyond these foundational practices was more limited and required collaboration leaders to seek out knowledge and resources on their own, which was often difficult within the context of the limited time and resources as discussed above.

**Collaboration Strategies.** Participants identified collaboration strategies such as cross-systems training as critical to forming a truly collaborative group, as this helped to increase understanding about the mental health system or the criminal legal system for collaboration members who are not employed in those systems. Despite bumping up against the criminal legal system, many mental health system workers know little about how it operates and vice versa. Increasing this understanding through trainings and in-services helped to promote the idea of a shared goal in which everyone across sectors was working to solve. Participants noted that education about the various systems involved in the collaboration helped to increase understanding of the roles each played in working towards a solution. Cross-systems training about the roles of other systems helped to produce a collaborative atmosphere between systems stakeholders. A participant thought increased understanding was foundational to their collaborative work,

There was no communication or true understanding of how both systems work together to treat your person who's involved, who's criminally justice involved, that…is straddling two sides. So, the step one to me, in terms of collaboration has been education. (Collaboration Leader 4, lines 48-51)

Participants also pointed to the importance of shared data and data-driven decision-making as important to prioritizing areas of focus, but also to monitor outcomes. However, participants described how most counties struggled with devising ways to share and manage data due to concerns about privacy and lack of capacity to fund or manage data. Correspondingly, some counties became creative in finding ways to use data to inform their CSCs, which included sharing qualitative data in terms of sharing success stories or in trying to figure out which agency had access to which data (data mapping).

## Discussion

As mental health-criminal legal cross-systems collaborations proliferate to develop solutions aimed at keeping people with serious mental illnesses in the community, it is increasingly critical to understand how they operate. We find evidence of several inner context, outer context, and bridging factors present, showing that the EPIS framework is applicable to mental health-criminal legal CSCs. As these groups are charged with implementing multiple programs and practices, we conceptualized the CSC itself not as a strategy to support a single agency to implement a program, but as the coordinating body charged with systems alignment and program implementation. As such, we explored the inner context of CSCs and not specific agencies. Concepts from the EPIS framework are revealed in our data with some similarity to the concepts as conceived in the original EPIS framework, albeit in a more complex fashion as the inner contextual factors represent a collaborative group comprised of multiple organizations across two or more systems instead of representing one organization charged with implementing an EBP.

Additionally, we identify several collaboration strategies at the systems level as important bridging factors contributing to systems alignment during the exploration and planning phases critical to the success of mental health-criminal legal CSCs. Therefore, the systems collaborative strategies, like engaging in Sequential Intercept Mapping, cross-systems training, or data sharing, are the glue that keeps members focused on problem areas and oriented to solutions. For example, participants shared that engaging in Sequential Intercept Mapping was helpful to jumpstarting their Stepping Up group. Likewise, cross-training for front line staff of mental health and criminal legal systems facilitated understanding of the roles of goals of each, leading to increased collaboration and shared programming. These systems-level collaboration strategies can contribute to a shared set of beliefs and values among multiple systems stakeholders, which may be key to the success of this type of collaborative group (Aarons et al., 2014b; Nowell, 2009).

Much of the research on collaboration strategies has focused on frontline or administrative collaboration strategies during the implementation phase (Bunger et al., 2024). Systems collaboration strategies during the exploration and planning phases may be critical to mental health-criminal legal collaborations that seek to make connections amongst systems stakeholders with different objectives. In an examination of mental health and criminal legal CSCs, Mackey and colleagues (2024) found that interagency collaboration, operationalized as coordination, integrated care, and data capacity, was favorably impacted by learning about and from other agencies and systems integration. Still, consensus-building activities had no impact on collaboration, suggesting that these relationships are nuanced. Data capacity was consistently cited as a barrier among our participants, due both to a lack of knowledge and skills and perceived as a barrier to making progress. A more rigorous assessment of collaborative strategies and their impact on CSCs, is needed to fully understand a distillation of the effective strategies to employ when starting, operating, and maintaining mental health-criminal legal CSCs. Such assessment is necessary especially when these inter-organizational factors are critical to implementation at the intersection of the mental health and criminal legal systems (Van Deinse et al., 2019a, 2019b).

Findings related to facilitators and barriers of CSCs shows which factors are important to mental health-criminal legal CSCs and the ways in which they operate to support or hinder collaborative efforts. Our analysis begins to develop an evidence base of inner, outer, and bridging factors important when convening CSCs charged with enacting systems-level change. Our analysis also conceptualizes the EPIS framework to account for the multiple levels of coordination and influence that exist when communities aim to work across systems silos. For example, leadership at two levels is very important to the operation of mental health-criminal legal CSCs as county leadership is important to garnering buy-in and participation across multiple sectors; whereas a coordinator-level position is important to guide the group and ensure their work progresses. Alignment between multiple levels of leadership may be critical to the implementation success (Aarons et al., 2014a), yet leadership has been understudied in CSCs. In some ways, these multiple leadership roles are akin to an agency CEO and supervisor needed to champion and implement, respectively, an evidence-based practice within an organization. For CSCs the reach and influence are greater, as these leaders corral multiple systems actors, perhaps requiring a different set of leadership skills.

We found that a lack of resources, including funding for the CSCs and capacity for data harmonization and management are often barriers to making progress in mental health-criminal justice CSCs. Since these collaborative efforts sit at the intersection of multiple sectors, there is typically not a single or direct funding source to support the collaborative group itself. Ideally, both mental health and criminal justice funds should be devoted to these efforts, but there may be barriers to blending funding from these different sectors. Leadership at county or state levels may need to organize efforts to devise funding streams for the CSCs, such as pooling funding to support designated staff time to lead the CSC that is not tied to a specific grant or program. This strategy was mentioned as a solution by participants from higher-resourced counties. Until then, local communities are left to creatively try to garner funding for efforts that are not clearly an intervention but likely to lead to systems change.

Likewise, the work of these CSCs may be undervalued or not well understood, and hence understaffed. Agencies may not be willing to devote dedicated staff and resources to these efforts until a clear plan is developed. Reluctance to dedicate resources, combined with the sense of voluntariness we heard expressed by participants, can contribute to a general lack of commitment among participants, thereby marginalizing individual agency involvement in the collaborative process undertaken by these CSC groups, further undermining the scope and reach of the CSC. Future research should explore these dynamics.

One limitation of this study is that it is focused on one state, Ohio, which may not represent all the ways that mental health-criminal legal cross-systems collaborations are organized throughout the country. In Ohio, awareness of and support for Stepping Up is spearheaded by a strong, statewide steering group led by a former state Supreme Court Justice who is widely respected and is a leader in a state-level task force focused on issues related to criminal justice and mental illness (Ohio Attorney General’s Office, 2025). Once counties decide to engage in Stepping Up, the steering group meets with stakeholders to educate and inform them of resources to support their efforts. Additionally, newly formed Stepping Up groups can engage in Sequential Intercept Mapping, which is paid for by the steering committee. The statewide practice of steering committee- funded Sequential Intercept Mapping likely contributed to our participants’ perspectives that most of the support for the CSCs is during the start-up phase, but not as much funding or support to sustain the group over time. While Stepping Up efforts are supported at the state level in some other states (e.g., Pennsylvania, Kansas), this seems to be more of an exception than the rule (The Stepping Up Initiative, 2025). Further, it should be noted that the views shared by participants of this qualitative study may not represent the perspective of other stakeholders involved in CSCs, either in Ohio or beyond. Future research may consider more targeted recruitment approaches to better understand how perceptions of CSCs may vary by social or geographic contexts. Finally, our study is neither an exploration of the internal processes not the outcomes of specific CSCs, as each participant was a representative from a different collaboration. Future research should explore the application of interorganizational frameworks to describe internal dynamics and processes of CSCs and how these processes may be linked with systems-level outcomes but these are excellent areas of inquiry for future studies.

There are several implications that can be drawn from our findings. At the policy-level, as more states and municipalities promote CSCs like Stepping Up, corresponding funding to provide dedicated staff and technical assistance should be allocated. Without funding many CSCs may not progress past the exploration stage, especially in rural or under-resourced counties. As mentioned above, it is not only important to describe the structure and function of CSCs but also to link collaboration strategies with outcomes. Relatedly, there is a need for leadership at both county and collaboration levels and communities would be wise to develop capacities in these areas. However, it is also important to not lose sight that the outcome is to improve the health and well-being of community members and not only to develop well-functioning groups.

The proliferation of mental health-criminal legal CSCs is likely both a response to the ongoing need to stop the overrepresentation of people with mental illnesses in the criminal legal system, but also likely the result of policy and practice levers attributed to Stepping Up and Sequential Intercept Mapping. They have the potential to solve some of the most intractable problems within the public behavioral health system over the past decades, yet little is known about how they operate and what impact these CSCs have. The EPIS framework has been useful in understanding how mental health-criminal legal CSCs are organized and what factors facilitate their formation and sustainability. Future research should investigate which factors are tied to the implementation of new practices, programs, and policies which in turn may improve the behavioral healthcare system and health outcomes for people with serious mental illnesses.
